# Diagnostic value of a ghrelin test for the diagnosis of GH deficiency after subarachnoid hemorrhage

**DOI:** 10.1530/EJE-13-0436

**Published:** 2013-10

**Authors:** K Blijdorp, L Khajeh, G M Ribbers, E M Sneekes, M H Heijenbrok-Kal, H J G van den Berg-Emons, A J van der Lely, F van Kooten, S J C M M Neggers

**Affiliations:** 1 Department of Medicine – EndocrinologyErasmus University Medical Center RotterdamPO Box 20403000 CA, RotterdamThe Netherlands; 2 Department of NeurologyErasmus University Medical Center RotterdamPO Box 20403000 CA, RotterdamThe Netherlands; 3 Department of Rehabilitation MedicineErasmus University Medical Center RotterdamPO Box 20403000 CA, RotterdamThe Netherlands; 4 Rijndam Rehabilitation CenterRotterdamThe Netherlands

## Abstract

**Objective:**

To determine the diagnostic value of a ghrelin test in the diagnosis of GH deficiency (GHD) shortly after aneurysmal subarachnoid hemorrhage (SAH).

**Design:**

Prospective single-center observational cohort study.

**Methods:**

A ghrelin test was assessed after the acute phase of SAH and a GH-releasing hormone (GHRH)–arginine test 6 months post SAH. Primary outcome was the diagnostic value of a ghrelin test compared with the GHRH–arginine test in the diagnosis of GHD. The secondary outcome was to assess the safety of the ghrelin test, including patients' comfort, adverse events, and idiosyncratic reactions.

**Results:**

Forty-three survivors of SAH were included (15 males, 35%, mean age 56.6±11.7). Six out of 43 (14%) SAH survivors were diagnosed with GHD by GHRH–arginine test. In GHD subjects, median GH peak during ghrelin test was significantly lower than that of non-GHD subjects (5.4 vs 16.6, *P*=0.002). Receiver operating characteristics analysis showed an area under the curve of 0.869. A cutoff limit of a GH peak of 15 μg/l corresponded with a sensitivity of 100% and a false-positive rate of 40%. No adverse events or idiosyncratic reactions were observed in subjects undergoing a ghrelin test, except for one subject who reported flushing shortly after ghrelin infusion.

**Conclusion:**

Owing to its convenience, validity, and safety, the ghrelin test might be a valuable GH provocative test, especially in the early phase of SAH.

## Introduction

The incidence of spontaneous subarachnoid hemorrhage (SAH) in The Netherlands varies between 5.7/100 000 subjects per year for men and 9.9/100 000 subjects per year for women. About 50% of SAH patients do not survive (1, 2). Those who do survive SAH have high rates of functional limitations that could lead to impaired quality of life, fatigue, decreased mobility, and loss of motivation. These symptoms could be caused by growth hormone deficiency (GHD) (3).

The prevalence of hypopituitarism after SAH varies between 0 and 55%, with GHD in 0–29% being the largest deficit among all SAH patients (3, 4, 5, 6, 7, 8, 9, 10, 11, 12). This neuro-endocrine dysfunction may result from the hypothalamic/pituitary system being damaged as a result of post hemorrhagic complications, e.g. local tissue pressure, toxic effects of the extravasated blood, ischemia, hydrocephalus, or local destruction during cerebral surgery.

GHD is diagnosed by a dynamic stimulatory test, as standard serum insulin-like growth factor 1 (IGF1) tests cannot discriminate between sufficient and insufficient GH secretion (13). Currently, the gold standard for the diagnosis of GHD is the insulin tolerance test. As this test is contra-indicated in the elderly and in patients with ischemic heart disease and seizures, it is regularly replaced by the GH-releasing hormone (GHRH)–arginine test, which is well-validated in adults (13). However, as both tests are burdensome and limited by side effects such as vasodilatation and paresthesia (14), they are not useful in the early phase after SAH where these side effects may be confused with the complications of SAH such as delayed cerebral ischemia which need proper treatment.

It might be possible to diagnose GHD occurring shortly after SAH by combining early hormonal screening with GH stimulation testing. By its binding to the GH secretagogue receptor-type 1a (GHSR-1a), ghrelin has a strong GH-releasing activity, and can be used as a diagnostic test. A ghrelin test is not limited by side effects and it has the advantage of also stimulating adrenocorticotrophin (ACTH) (15). As there is little data describing the use of ghrelin as a GH-stimulating diagnostic test, the aim was to determine the diagnostic value of a ghrelin test shortly after SAH to identify subjects with GHD and to define the cutoff limit of the GH peak below which GHD is confirmed.

## Subjects and methods

### Study design

This study was part of the HIPSS (hypopituitarism in patients after subarachnoid hemorrhage study), a prospective single-center observational cohort study at the Erasmus University Medical Center Rotterdam. It was approved by the Local Medical Ethical Committee (METC). All patients gave written informed consent. Adverse events were registered and reported to the Central Committee for human scientific research (CCMO).

### Subjects

Included in this study were all patients with aneurysmal SAH, aged ≥18 years. All patients included in this study were treated for SAH before inclusion. They were dismissed from the intensive care unit (ICU) and admitted to the Department of Neurology of the Erasmus Medical Center between June 2009 and June 2012. We excluded patients with any hypothalamic/pituitary disease, former cranial irradiation, prior significant head trauma, or any other medical condition or laboratory abnormality that may have interfered with the outcome of the study.

### Clinical definitions and outcome measures

The primary outcome was to compare the ghrelin test with the GHRH–arginine test in terms of their value for the diagnosis of GHD. The secondary outcome was to assess the safety of the ghrelin test, including patients' comfort, adverse events, and idiosyncratic reactions.

The diagnosis of SAH was confirmed by computerized tomography (CT) or lumbar puncture. Localization of the aneurysm was determined by CT angiography or a digital subtraction angiography. We defined GH deficiency (GHD) as an insufficient GH response to a GHRH–arginine test, assessed at 6 months after SAH. We used the following cutoff limits to define GHD for the GHRH–arginine test: for subjects with a BMI <25 kg/m^2^, a peak GH <11 μg/l; for subjects with a BMI between 25 and 30 kg/m^2^, a peak GH <8 μg/l; and for subjects with a BMI >30 kg/m^2^, a peak GH <4 μg/l (13).

To rule out interference of other hormonal deficiencies, we simultaneously measured basal hormone levels including cortisol, free thyroxin, thyroid-stimulating hormone, prolactin (PRL), IGF1, and IGF binding protein 3, in men and women; testosterone in men; and estradiol, follicle-stimulating hormone, and luteinizing hormone in women. IGF1 was assessed by immulite 2000 (DPC Biermann GmbH/Siemens, Fernwald, Germany), a solid-phase, enzyme-labeled chemiluminescent immunometric assay, with an intra-assay variability of 2–5%, and an inter-assay variability of 3–7% (16), we calculated IGF1 mean SDS (17). IGFSDS are depicted in Table 1.

### Acute phase

A ghrelin test was assessed in a fasting patient at rest, during admission after SAH. BMI was calculated from height and body weight (18). At baseline, we measured GH and cortisol, and then infused 1 μg/kg body weight acylated ghrelin. GH and cortisol were measured after 30 and 60 min. A recent study has shown that individual peak GH response to ghrelin occurred in all subjects between 15 and 45 min with a curve maximum at 30 min after ghrelin test, in GHD and non-GHD patients (19).

### Six months post-SAH

A GHRH–arginine test was assessed at the research unit 6 months after SAH. GH was measured, followed by infusion of 1 μg/kg body weight GHRH and 0.5 mg/kg arginine within 30 min. GH was assessed every 5–15 min.

### Statistical analysis

Data were expressed as mean±s.d. for normal distributed variables or as median (ranges) for nonnormative variables. To evaluate differences in GH peak between GHD and non-GHD subject, Mann–Whitney *U* test was assessed. To determine the value of applying a ghrelin test shortly after SAH to identify subjects with GHD, the sensitivity, specificity, and positive and negative predictive values were calculated using receiver operating characteristics (ROC) analysis. The reference test for the detection of GHD is the GHRH–arginine test. In addition, the likelihood ratios of a positive ghrelin test and of a negative ghrelin test were calculated. All statistical analyses were performed with SPSS version 20.0 (SPSS, Inc., Chicago, IL, USA).

## Results

### Patients

**Figure 1:**
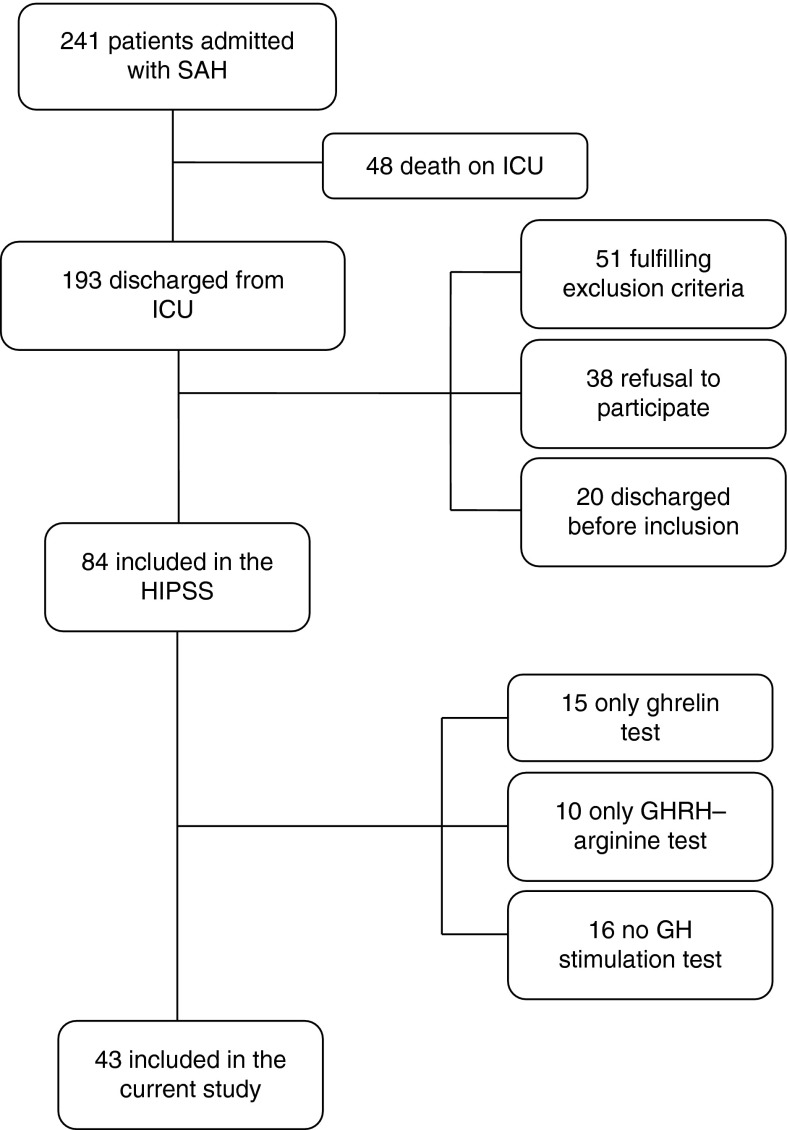
Inclusion criteria for subjects. SAH, subarachnoid hemorrhage; ICU, intensive care unit; GH, growth hormone; GHRH, GH-releasing hormone; HIPSS, hypopituitarism in patients after subarachnoid hemorrhage study.

Out of 241 patients admitted to the ICU with the diagnosis of SAH, 193 survived. Fifty-one patients did not fulfill the inclusion criteria, 38 refused to participate, and 20 patients were discharged before the inclusion was fulfilled. Eventually, 84 patients were included in the HIPSS study (Fig. 1). Fifteen patients had a ghrelin test and no GHRH–arginine test. They found the endurance of the dynamic tests too strenuous. At the start of the study, ghrelin test was not at our disposal; so ten patients only had a GHRH–arginine test but no ghrelin test, and 16 patients did not give us permission to perform the dynamic tests.

In 43 patients, both the ghrelin test and the GHRH–arginine test were assessed and therefore the data of 43 patients were analyzed in the current study. Patient characteristics are outlined in Table 1. Median time between occurrence of SAH and ghrelin test was 18 days. Patient characteristics of excluded and included subjects did not differ significantly (sex: male, 9/41 (22%) vs 15/43 (35%), *P*=0.142; mean age, 56.3 vs 56.6 years, *P*=0.912).

### Outcome

**Figure 2:**
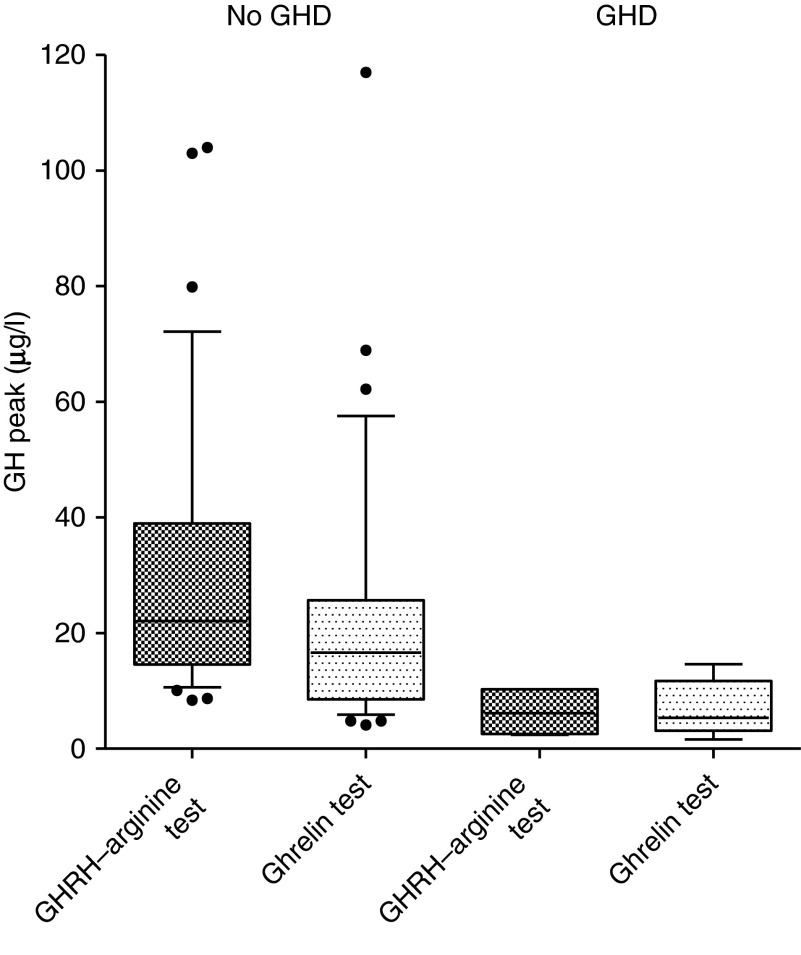
GH peak in GH-deficient subjects vs non-GH-deficient subjects. GH, growth hormone; GHRH, GH-releasing hormone.

Six out of 43 (14%) SAH survivors were diagnosed with GHD by the GHRH–arginine test. The median GH peak of GHD subjects was 5.4 μg/l during the ghrelin test and 6.2 μg/l during the GHRH–arginine test (Fig. 2). In GHD subjects, median GH peak during ghrelin test was significantly lower than that of non-GHD subjects (5.4 (range 1.6–14.6) vs 16.6 (4.1–117), *P*=0.002). We observed a low cortisol level in one patient (2%) during ghrelin testing. This subject who was re-tested using the synacthen-test did not reveal a secondary hypocortisolaemia. Three male patients with GHD also had a hypogonadotropic hypogonadism. Another nine patients in this study had hypogonadotropic hypogonadism after 6 months.

ROC analysis showed an area under the curve of 0.869. Table 2 gives an overview of sensitivity, specificity, positive predictive value, and negative predictive value for different cutoff limits of the GH peak during a ghrelin test. A sensitivity of 100%, which is needed to diagnose every GHD subject, gives a false-positive rate of 40% (1-specificity), belonging to a cutoff of 15 μg/l (Table 2). A cutoff of 11.4 μg/l gives the optimal combination of the highest sensitivity (83%) and the highest specificity (73%).

### Adverse events/safety measures

Only one subject reported an adverse effect, i.e. flushing starting shortly after ghrelin infusion, which lasted for 30 min. During this period, we monitored heart frequency and blood pressure, which remained stable. No adverse events or idiosyncratic reactions were reported by or observed in the other subjects undergoing a ghrelin test.

## Discussion

Ghrelin testing is a safe and valid alternative in the diagnosis of GHD shortly after SAH. Other current available GH provocative tests might be burdensome and their use is limited in SAH survivors because of their possible side effects.

Ghrelin, a 28-amino-acid peptide hormone, is predominantly produced by the stomach, stimulates food intake and a positive energy balance, and plays an important role in fat metabolism (20). Besides, ghrelin has strong GH-releasing activity by binding to the GHSR-1a. Apart from stimulating GH secretion, ghrelin exhibits hypothalamic activities resulting in the stimulation of PRL and ACTH secretion (20). Previous studies described the use of ghrelin as a GH stimulation test, when combined with GHRH, illustrating a strong and potent GH-releasing activity due to the synergistic effects of ghrelin and GHRH (21, 22, 23, 24). In addition, administration of ghrelin alone illustrated a GH release with a clear dose–response curve in normal subjects (22, 25). In GHD subjects, GH response was significantly lower than that in normal subjects, illustrating the value of a ghrelin test in diagnosing GHD (24). The use of GH-releasing peptide 6, which is an artificial hexapeptide activating the GHSR1a, in combination with GHRH, has been described as a potent GH-provocative test in several studies investigating pituitary dysfunction after traumatic brain injury (26, 27, 28). In the current study, a ghrelin test was assessed shortly after SAH and illustrated a significantly lower GH peak in GHD subjects compared with non-GHD subjects. ROC analysis showed a high accuracy of the ghrelin test when compared with GHRH–arginine test. The GHRH–arginine test, however, is inconvenient in SAH patients because of its known side effects such as vasodilatation and paresthesia (14). A more safe and patient friendly test, like a ghrelin test, would be preferred, especially in disabled patients who are critically ill. Another known side effect of GHRH administration is transient facial flushing, which occurs in 25% of the patients (14). In our view, this side effect might be related to fluctuating blood pressure, which is also undesirable in patients in the early phase after SAH.

In a recent paper published by Gasco *et al*. (19), ghrelin test proved to be a valid diagnostic test for GHD in adults with reliable cutoff limits in lean and overweight patients. Furthermore, they concluded that obesity has an impact on the GH response to ghrelin, lowering the predictive value of the test in these patients, so caution must be taken when it comes to interpreting ghrelins test in obese patients. However, obesity has an impact on almost all dynamic GHD tests (29). They also described that facial flushing occurred in a smaller group of patients after ghrelin test compared with GHRH–arginine test. Other adverse events of ghrelin administration have not been described so far except for patients receiving more than 250 μg of ghrelin, who reported transient hyperhydrosis (22, 25).

Beside its convenience, the ghrelin test would be preferred above the GHRH–arginine test because, by stimulating ACTH release, adrenal function could be evaluated. The prevalence of adrenocorticotropic deficiency following SAH varies from 0 to 40% (6, 7, 8, 12) and could lead to life-threatening conditions if untreated. This elucidates added value of the ghrelin test as it can be used to detect a secondary adrenocorticotropic deficiency early after SAH. However, as the ACTH-releasing activity is less potent, higher ghrelin doses may be needed in order to discriminate between sufficient and insufficient cortisol response (24). Therefore, the evaluation of adrenal function by ghrelin testing should be elucidated in further studies.

We observed that a second confirmatory test was needed to confirm true GHD in patients who had low GH response to the ghrelin test. A recent study by Gardner et al. (30) also concluded that real GHD in SAH patients has to be confirmed by two different stimulation tests and by the application of BMI specific cutoffs. Based on these findings, we recommend the usage of two different stimulation tests for the confirmation of true isolated GHD.

The limitation of this study was that cutoff limits for the ghrelin test could not be defined, as only six patients had been diagnosed with GHD. Fortunately, the cut off values for ghrelin test have been recently published by a different group of investigators (19).

Secondly, the time between GH stimulation tests could be an important confounder because hypopituitarism in the acute phase has been described to resolve in the post-acute phase (11, 31). This might be caused by the SAH or SAH's related complications. Furthermore, the fact that the subjects are critically ill could also have a significant impact on the results of the ghrelin test. However, some studies have shown that in contrast to both hypogonadism and hypocortisolism that can normalize during follow-up, GHD prevalence remained stable until 1 year after SAH (4, 32). Ideally, multiple GHD tests in this early phase could exclude these possible confounders; however, it is not feasible to perform multiple tests in critically ill SAH patients.

In conclusion, the ghrelin test discriminated between GHD and non-GHD survivors of SAH and displayed a high accuracy compared with the GHRH–arginine test. Owing to its convenience, validity, and safety, the ghrelin test is a novel GH provocative test that can be used as a screening tool for the prediction of late GHD in SAH patients.

## Figures and Tables

**Table 1:** Baseline characteristics and results of dynamic tests of included and excluded survivors.

	**Values are expressed as *n* (%) of subjects**^a^	
	SAH patients with ghrelin test and GHRH–arginine test (*n*=43)	Excluded SAH patients in the current study (*n*=41)
Sex (male)	15 (35)	9 (22)
Age (years)	56.6±11.7	56.3±11.8
BMI	25.3±3.1	24.8±4.2
<25 kg/m^2^	22 (51)	23 (56)
25–30 kg/m^2^	17 (40)	12 (29)
>30 kg/m^2^	4 (9)	6 (15)
WFNS		
I	17 (40)	21 (51)
II	15 (35)	10 (24)
III	3 (7)	0
IV	5 (11)	6 (15)
V	3 (7)	4 (10)
Location of aneurysm		
Anterior circulation	24 (56)	25 (61)
Posterior circulation	19 (44)	16 (39)
Aneurysm treatment		
Endovascular treatment	37 (86)	29 (71)
Clipping	6 (14)	11 (27)
None	0	1 (2)
Hydrocephalus	19 (44)	13 (32)
Delayed cerebral ischemia	3 (7)	5 (12)
GH peak ghrelin test (μg/l)	15.3 (1.6–117)^b^	*n*=15, 19.3 (8.2–35.4)^b^
GH response <5 μg/l	6 (14)	0
Baseline IGF1 SDS (95% CI)	−0.06 (−2.1; 1.94)^c^	
GH peak GHRH–arginine test (μg/l)	18.1 (2.4–104)^b^	*n*=10, 23.2 (4.6–114.0)^b^
Insufficient GH response	6 (14)	2 (5)
6-month IGF1 SDS (95% CI)	−0.7 (−2.56; 1.16)^c^	

GH, growth hormone; GHRH, GH-releasing hormone; SAH, subarachnoid hemorrhage; WFNS, World Federation of Neurologic Surgeons grading system for subarachnoid haemorrhage scale.

a Unless otherwise specified.

b Expressed as median (range).

c Expressed as mean (95% CI).

**Table 2:** Summary statistics for various GH cutoff values for the ghrelin test predicting GH deficiency.

**GH cutoff for ghrelin test**	**Sensitivity** (%)	**Specificity** (%)	**PPV** (%)	**NPV** (%)
2.6	17	100	100	88
3.9	33	100	100	90
4.8	50	97	75	92
6.1	67	92	57	94
11.4	83	73	33	96
15.0	100	60	29	100
20.0	100	38	21	100
30.4	100	16	16	100

GH, growth hormone; PPV, positive predictive value; NPV, negative predictive value.
